# Supplementary material for: FERN – a Java framework for stochastic simulation and evaluation of reaction networks
Source: BMC Bioinformatics. 2008 Aug 29;9:356. doi: 10.1186/1471-2105-9-356 (PMC2553347; doi:10.1186/1471-2105-9-356)
Supplement: Additional file 1 — FERN distribution, Version 1.3. This archive contains the FERN source code and binaries as well as documentation and example models in FernML and SBML. [file 1471-2105-9-356-S1.zip › fern/doc/javadoc/fern/tools/gnuplot/class-use/GnuPlot.html]

Uses of Class fern.tools.gnuplot.GnuPlot


---


|  |  |  |
| --- | --- | --- |
| **Overview** \| **Package** \| **Class** \| **Use** \| **Tree** \| **Deprecated** \| **Index** \| **Help** | |  |
| PREV   NEXT | **FRAMES**    **NO FRAMES**     **All Classes** |  |


---


## **Uses of Class fern.tools.gnuplot.GnuPlot**

| Packages that use GnuPlot | |
| --- | --- |
| **fern.benchmark** | Provides classes for benchmarking simulations (time benchmarks, histogram distance calculation). |
| **fern.simulation.observer** | Provides classes to observe certain aspects of simulations. |
| **fern.tools.gnuplot** | Provides classes to use gnuplot from java. |

Uses of GnuPlot in fern.benchmark

| Methods in fern.benchmark that return GnuPlot | |
| --- | --- |
| `GnuPlot` | `Benchmark.toGnuplot(GnuPlot gnuplot, String[] dataLabels, String[] styles)`             Adds the benchmark data without conversion to a given `GnuPlot` object. |
| `GnuPlot` | `Benchmark.toGnuplot(String[] dataLabels, String[] styles)`             Adds the benchmark data without conversion to a new `GnuPlot` object. |
| `GnuPlot` | `Benchmark.toGnuPlotAsHistogram(GnuPlot gnuplot, String[] dataLabels, String[] styles)`             Adds the benchmark data as histogram to a given `GnuPlot` object. |
| `GnuPlot` | `Benchmark.toGnuPlotAsHistogram(String[] dataLabels, String[] styles)`             Adds the benchmark data as histogram to a new `GnuPlot` object. |

| Methods in fern.benchmark with parameters of type GnuPlot | |
| --- | --- |
| `GnuPlot` | `Benchmark.toGnuplot(GnuPlot gnuplot, String[] dataLabels, String[] styles)`             Adds the benchmark data without conversion to a given `GnuPlot` object. |
| `GnuPlot` | `Benchmark.toGnuPlotAsHistogram(GnuPlot gnuplot, String[] dataLabels, String[] styles)`             Adds the benchmark data as histogram to a given `GnuPlot` object. |

Uses of GnuPlot in fern.simulation.observer

| Methods in fern.simulation.observer that return GnuPlot | |
| --- | --- |
| `GnuPlot` | `RandomNumberGeneratorCallObserver.toGnuplot()` |
| `GnuPlot` | `LeapObserver.toGnuplot()` |
| `GnuPlot` | `IntervalObserver.toGnuplot()` |
| `GnuPlot` | `GnuPlotObserver.toGnuplot()`             Creates a new `GnuPlot` object and passes the actual observer data to it. |
| `GnuPlot` | `FireTypeObserver.toGnuplot()` |
| `GnuPlot` | `AmountAtMomentObserver.toGnuplot()` |
| `GnuPlot` | `RandomNumberGeneratorCallObserver.toGnuplot(GnuPlot gnuplot)` |
| `GnuPlot` | `LeapObserver.toGnuplot(GnuPlot gnuplot)` |
| `GnuPlot` | `IntervalObserver.toGnuplot(GnuPlot gnuplot)` |
| `GnuPlot` | `GnuPlotObserver.toGnuplot(GnuPlot gnuplot)`             Passes the actual observer data to a `GnuPlot` object. |
| `GnuPlot` | `FireTypeObserver.toGnuplot(GnuPlot gnuplot)` |
| `GnuPlot` | `AmountAtMomentObserver.toGnuplot(GnuPlot gnuplot)` |
| `GnuPlot` | `IntervalObserver.toGnuplotRecent()`             Creates a new `GnuPlot` object and passes the recent observer data to it. |
| `GnuPlot` | `IntervalObserver.toGnuplotRecent(GnuPlot gnuplot)`             Passes the recent observer data to a `GnuPlot` object. |

| Methods in fern.simulation.observer with parameters of type GnuPlot | |
| --- | --- |
| `GnuPlot` | `RandomNumberGeneratorCallObserver.toGnuplot(GnuPlot gnuplot)` |
| `GnuPlot` | `LeapObserver.toGnuplot(GnuPlot gnuplot)` |
| `GnuPlot` | `IntervalObserver.toGnuplot(GnuPlot gnuplot)` |
| `GnuPlot` | `GnuPlotObserver.toGnuplot(GnuPlot gnuplot)`             Passes the actual observer data to a `GnuPlot` object. |
| `GnuPlot` | `FireTypeObserver.toGnuplot(GnuPlot gnuplot)` |
| `GnuPlot` | `AmountAtMomentObserver.toGnuplot(GnuPlot gnuplot)` |
| `GnuPlot` | `IntervalObserver.toGnuplotRecent(GnuPlot gnuplot)`             Passes the recent observer data to a `GnuPlot` object. |

Uses of GnuPlot in fern.tools.gnuplot

| Methods in fern.tools.gnuplot with parameters of type GnuPlot | |
| --- | --- |
| `void` | `GnuPlot.merge(GnuPlot gnuplot)`             Merges another gnuplot object with this one by add its axes and copy its default style to the axes (if they have no style). |

---


|  |  |  |
| --- | --- | --- |
| **Overview** \| **Package** \| **Class** \| **Use** \| **Tree** \| **Deprecated** \| **Index** \| **Help** | |  |
| PREV   NEXT | **FRAMES**    **NO FRAMES**     **All Classes** |  |


---
